# Differential host susceptibility and bacterial virulence factors driving Klebsiella liver abscess in an ethnically diverse population

**DOI:** 10.1038/srep29316

**Published:** 2016-07-13

**Authors:** I. Russel Lee, James S. Molton, Kelly L. Wyres, Claire Gorrie, Jocelyn Wong, Chu Han Hoh, Jeanette Teo, Shirin Kalimuddin, David C. Lye, Sophia Archuleta, Kathryn E. Holt, Yunn-Hwen Gan

**Affiliations:** 1 Department of Biochemistry, Yong Loo Lin School of Medicine, National University of Singapore; 2 Division of Infectious Diseases, University Medicine Cluster, National University Health System, Singapore; 3 Department of Medicine, Yong Loo Lin School of Medicine, National University of Singapore; 4 Centre for Systems Genomics, University of Melbourne, Parkville, Victoria 3010, Australia; 5 Department of Biochemistry and Molecular Biology, Bio21 Molecular Science and Biotechnology Institute, University of Melbourne, Parkville, Victoria 3010, Australia; 6 Department of Laboratory Medicine, Microbiology Unit, National University Hospital, Singapore; 7 Department of Infectious Diseases, Singapore General Hospital, Singapore; 8 Communicable Disease Center, Institute of Infectious Diseases and Epidemiology, Tan Tock Seng Hospital, Singapore

## Abstract

Hypervirulent *Klebsiella pneumoniae* is an emerging cause of community-acquired pyogenic liver abscess. First described in Asia, it is now increasingly recognized in Western countries, commonly afflicting those with Asian descent. This raises the question of genetic predisposition versus geospecific strain acquisition. We leveraged on the Antibiotics for Klebsiella Liver Abscess Syndrome Study (A-KLASS) clinical trial ongoing in ethnically diverse Singapore, to prospectively examine the profiles of 70 patients together with their isolates’ genotypic and phenotypic characteristics. The majority of isolates belonged to capsule type K1, a genetically homogenous group corresponding to sequence-type 23. The remaining K2, K5, K16, K28, K57 and K63 isolates as well as two novel *cps* isolates were genetically heterogeneous. K1 isolates carried higher frequencies of virulence-associated genes including *rmpA* (regulator of mucoid phenotype A), *kfu* (Klebsiella ferric uptake transporter), *iuc* (aerobactin), *iro* (salmochelin) and *irp* (yersiniabactin) than non-K1 isolates. The Chinese in our patient cohort, mostly non-diabetic, had higher prevalence of K1 infection than the predominantly diabetic non-Chinese (Malays, Indian and Caucasian). This differential susceptibility to different capsule types among the various ethnic groups suggests patterns of transmission (e.g. environmental source, familial transmission) and/or genetic predisposition unique to each race despite being in the same geographical location.

A new, hypervirulent variant of *Klebsiella pneumoniae* (hvKP) has been increasingly described in the last two decades. Combinations of clinical as well as bacterial genotypic and phenotypic features distinguish this variant from the “classic” opportunistic *K. pneumoniae*. The first is its ability to cause life-threatening disease in healthy individuals, although diabetes mellitus is a risk factor^1^,^2^,^3^,^4^. Unique invasive syndromes include community-acquired monomicrobial pyogenic liver abscess, and a propensity for metastatic spread to unusual distant sites including the eye, brain and lung^2^,^4^,^5^. The second is its ability to more efficiently acquire iron, and enhanced ability to produce the polysaccharide capsule that typically results in the hypermucoviscous phenotype^5^,^6^,^7^,^8^. At least 79 capsule types of *K. pneumoniae* exist^9^. For hvKP, eight types have been described to date: K1, K2, K5, K16, K20, K54, K57 and KN1^1^,^3^,^10^,^11^. The vast majority of hvKP that cause Klebsiella liver abscess (KLA) are K1 or K2 capsule types^3^,^4^,^12^. Through multi-locus sequence typing (MLST), sequence-type (ST) 23 was found to be strongly associated with capsule type K1, while ST25, ST86, ST375 and ST380 have been associated with capsule type K2^13^,^14^,^15^,^16^.

Although the route of hvKP entry into humans has not been established, the gastrointestinal tract appears to be the dominant site of colonization. *K. pneumoniae* isolated from healthy carriers has identical pulsed-field gel electrophoresis profile as well as similar virulence-associated genes and median lethal dose values in mouse lethality assays as hvKP isolated from patients with liver abscess^17^. When mice were orally infected with hvKP, four distinct stages were observed to develop sequentially: intestinal colonization, extraintestinal dissemination, hepatic replication and septic metastasis^18^.

While hvKP was first discovered in the Asia Pacific Rim (e.g. Taiwan) in the mid-1980s^19^, it is now increasingly recognized in Western countries (e.g. North America)^15^,^20^,^21^. However, most cases acquired in Western countries commonly occur among Asians^1^,^2^. Inevitably, travel to the Asia Pacific Rim or exposure to people from that region is assumed to increase the risk of KLA. Indeed, hvKP strain acquisition resulting in colonization and subsequent infection from international travel has been documented^22^,^23^. Nonetheless, this risk factor is unable to account for all the KLA cases occurring outside of Asia^15^,^20^. This raises the question about the importance of host genetic susceptibility (Asians, particularly of Chinese ethnicity) versus geographically defined pathogen exposure and acquisition (Asia Pacific Rim) in driving the epidemiology of KLA. Research on hvKP is most extensive in Asia. Various associations of patient clinical features and bacterial molecular characteristics influencing KLA development have been reported from Taiwan, China, Hong Kong and Korea^3^,^4^,^16^,^24^,^25^. However, these clinical studies of large case series were almost exclusively conducted on ethnically homogenous populations. Therefore, the issue of genetic predisposition versus geospecific strain acquisition remains unresolved.

We leveraged on a prospective multi-center randomized clinical trial [Antibiotics for Klebsiella Liver Abscess Syndrome Study (A-KLASS)] ongoing in Singapore, where KLA is now the leading cause of liver abscess^26^. Singapore is a multiracial and multicultural country, consisting of 76.2% Chinese, 15% Malay, 7.4% Indian, and 1.4% other races^27^. Information on the prevalence of diabetes mellitus among the various ethnic groups is also available: 17.2% of Indians, 16.6% of Malays and 9.7% of Chinese have underlying diabetes^28^. In order to understand what drives the development of KLA, we collected bacterial isolates from 70 consecutive A-KLASS participants of all four ethnic groups, with information on their clinical profile and parameters. We explored the relationship between clonal distribution and virulence profiles of KLA isolates with the corresponding patient demographics and host susceptibility factors.

## Results

### KLA patient characteristics

Seventy participants were recruited between 7 March 2013 and 19 October 2015. Patient characteristics are summarized in Table 1. The mean age was 59.8 ± 11.9 years. Eighty percent (56/70) were male. Seventy-six percent (53/70) were Chinese, 21.4% (15/70) Malay, 1.4% (1/70) Indian, and 1.4% (1/70) Caucasian. Compared to Singapore’s ethnic distribution, Malays were slightly over-represented in our patient cohort with Indians under-represented, whereas Chinese and other races were appropriately represented. Type 2 diabetes mellitus was the most frequent comorbidity (47.1%, 33/70). Other common comorbidities included cardiovascular disease (41.4%, 29/70), hypertension (34.3%, 24/70) and hyperlipidemia (31.4%, 22/70). Forty percent (28/70) had multiloculated liver abscesses, and the mean abscess diameter was 57.6 ± 30.6 mm.

### Genotyping of clinical isolates

To identify the different capsule types among KLA strains isolated from these 70 patients, we genotyped the capsular polysaccharide synthesis (*cps*) genes via PCR-based *wzy/wzx-*typing (*n* = 70) and whole-genome derived *wzi-*typing (*n* = 27). The genotypic and phenotypic virulence profiles of all 70 isolates are summarized in Table 2. Capsule types were successfully assigned for 68 isolates. No previously defined *cps* loci were detected for isolates TTSH04 and NUH11, but subsequent genomic screens indicated the presence of novel *cps* loci in both cases, which likely correspond to novel capsule types. The *cps* locus in TTSH04 showed similarity to that of isolate BIDMC25 in the *K. pneumoniae* BIGS database (http://www.ncbi.nlm.nih.gov/bioproject/202036). The phenotypic capsule type of BIDMC25 is unknown, and its full-length *cps* locus does not match any of those of the 79 published references^9^. Overall, capsule type K1 was the most common (64.3%, 45/70), followed by K2 (20%, 14/70), K5 (5.7%, 4/70) and K57 (2.9%, 2/70). K16, K28, K63 and the two novel *cps* loci were detected in one isolate each.

We further screened isolates for possession of acquired virulence genes associated with iron metabolism including *kfu* (ferric uptake transporter), *iuc* (aerobactin siderophore), *iro* (salmochelin siderophore) and *irp* (yersiniabactin siderophore). We also screened for the three known genomic copies of *rmpA* (regulator of mucoid phenotype A; transcriptional activator of *cps* genes) and expression of the hypermucoviscous phenotype. All the K1 isolates were positive for *kfu, iuc* and *iro,* whereas 93.3% (42/45) were positive for *irp.* All the K1 isolates were hypermucoviscous, with 97.8% (44/45) harboring two copies each of *rmpA* while one other isolate had a single copy. Thirteen of these K1 isolates, including one representative *irp-*negative isolate, were selected for whole-genome sequencing (WGS), which confirmed the presence of the complete sequences of the virulence-related clusters detected by PCR. Subsequent *in silico* MLST analysis revealed that all 13 isolates represented ST23 (Supplementary Table S1). Taken together, these findings suggest that the 45 K1 isolates are closely related, and all are likely to represent the ST23-K1 lineage.

In contrast, there was diversity of virulence gene content among the 14 K2 isolates. Thirty-six percent (5/14) were positive for *kfu,* 64.3% (9/14) were positive for *irp,* 92.9% (13/14) were positive for *iuc,* and 100% (14/14) were positive for *iro*. While 92.9% (13/14) were hypermucoviscous, there was variation in *rmpA* copy number: 35.7% (5/14), 57.1% (8/14) and 7.1% (1/14) harbored two, one and zero copies of *rmpA*, respectively. The one particular K2 isolate (named NUH04) that lacked *rmpA* was non-mucoviscous, consistent with *rmpA* regulation of the hypermucoviscous phenotype. Concordant with the PCR results, analysis of the whole genomes of five randomly selected K2 isolates showed that they each represented a distinct MLST profile (ST65, ST373, ST380, ST2038 and ST2039), highlighting the genotypic diversity of the K2 isolates (Supplementary Table S1). Of particular note, two of the K2-associated STs were newly identified in this study: ST2039 is the non-mucoviscous NUH04 strain, while ST2038 (named NUH14) is a double-locus variant of ST23. Interestingly, NUH14 was the only non-K1, non-ST23 isolate that tested PCR-positive for *allS* (activator of allantoin metabolism), which was carried by the other 45 K1 isolates. Therefore, NUH14 may share recent ancestry with the ST23 lineage.

**Figure 1:**
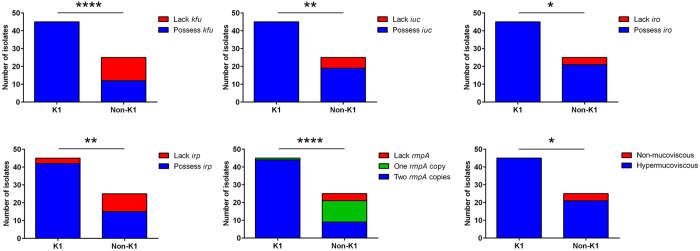
K1 isolates carried higher frequencies of virulence-associated genes than non-K1 isolates. These include *kfu* (100% vs 48%; *P* < 0.0001 by Fisher’s exact test), *iuc* (100% vs 76%; *P* = 0.001 by Fisher’s exact test), *iro* (100% vs 84%; *P* = 0.014 by Fisher’s exact test), *irp* (93.3% vs 60%; *P* = 0.001 by Fisher’s exact test) and *rmpA* (mean copy number: 1.98 vs 1.20; *P* < 0.0001 by Student’s *t* test). Expression of the hypermucoviscous phenotype correlated with possession of at least one *rmpA* copy in the genome.

We also sequenced the whole genomes of three K5 isolates, one K16 isolate, one K28 isolate, one K57 isolate and one K63 isolate, as well as both of the novel *cps* isolates. Each of these nine isolates represented a distinct ST, including another newly identified one, ST2037, belonging to the novel *cps* isolate TTSH04 (Supplementary Table S1). Consistent with the WGS data, PCR screening revealed diversity of virulence gene content among the non-K1/K2 isolates. Fifty-five percent (6/11) were positive for *iuc* and *irp,* and 63.6% (7/11) were positive for *kfu* and *iro*. Only 72.7% (8/11) were hypermucoviscous, and there was variation in *rmpA* copy number: 36.4% (4/11), 36.4% (4/11) and 27.3% (3/11) harbored two, one and zero copies of *rmpA*, respectively. The three isolates (K28 isolate NUH29, novel *cps* isolates TTSH04 and NUH11) that did not possess *rmpA* were non-mucoviscous, again consistent with *rmpA* regulation of the hypermucoviscous phenotype. Taken together, the non-K1 isolates are genetically diverse whereas the K1 isolates likely represented a single clone (ST23), consistent with previous studies^13^,^15^,^29^. Between these two distinct groups, clonal K1 isolates carried significantly higher frequencies of virulence-associated genes (*kfu, iuc, iro, irp* and *rmpA*) compared to non-clonal, non-K1 isolates (Fig. 1).

### Resistance to human serum

**Figure 2:**
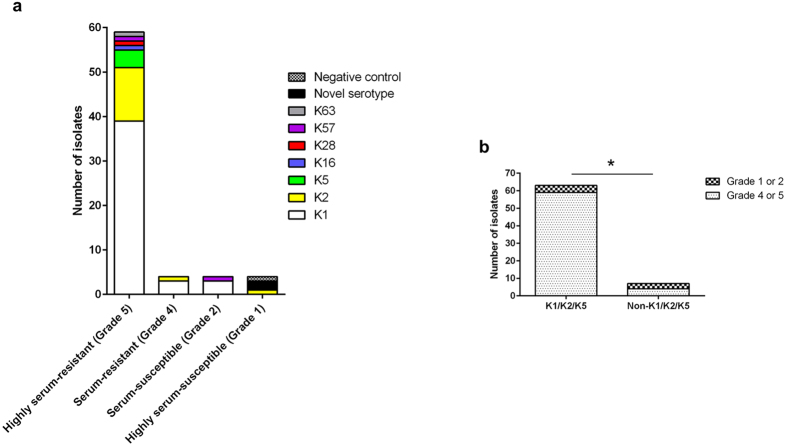
K1/K2/K5 isolates were more resistant to serum exposure than non-K1/K2/K5 isolates. (**a**) Serum resistance level of isolates from the different capsule types. Responses were graded as follows: grade 5 (highly serum-resistant), viable CFU after 3 hours of incubation in serum > 100% of the inoculum; grade 4 (serum-resistant), 71– 100%; grade 2 (serum-susceptible), 1–30%; grade 1 (highly serum-susceptible), 0%. The negative control, *E. coli* OP50, was highly-serum susceptible. (**b**) Comparison of prevalence of serum-resistant (grade 4 or 5) population between K1/K2/K5 isolates and non-K1/K2/K5 isolates (93.7% vs 57.1%; *P* = 0.019 using Fisher’s exact test).

The majority (90%, 63/70) of the isolates were resistant to pooled healthy human serum (Fig. 2a). This is generally consistent with our finding that 94.3% (66/70) of the isolates expressed the hypermucoviscous phenotype, which confers protection against the bactericidal effects of serum^1^,^2^,^30^. Specifically, 59/70 isolates were highly serum-resistant including one non-mucoviscous K28 strain NUH29, 4/70 isolates were serum-resistant, 4/70 isolates were serum-susceptible, and 3/70 isolates were like the non-pathogenic *Escherichia coli* OP50 that is highly serum-susceptible. The vast majority (93.7%, 59/63) of K1/K2/K5 isolates were classified as either highly serum-resistant or serum-resistant, compared to only 57.1% (4/7) of non-K1/K2/K5 isolates (*P* = 0.019; Fig. 2b). Amongst the non-K1/K2/K5 isolates, there was no association between virulence gene content and serum resistance. Therefore our data support an association between capsule types K1, K2 and K5 with the hypermucoviscous and serum-resistant phenotypes, which in turn are associated with increased virulence potential^31^,^32^.

### Ethnic susceptibility to capsule types

**Figure 3:**
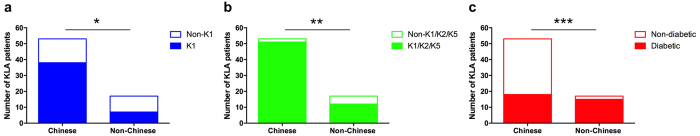
The predominantly non-diabetic Chinese were more likely to be infected by K1 isolates and less likely to be infected by uncommon KLA capsule types (non-K1/K2/K5) than the predominantly diabetic non-Chinese. (**a**) Comparison of prevalence of K1 infection between Chinese and non-Chinese (71.7% vs 41.2%; *P* = 0.040 by Fisher’s exact test). (**b**) Comparison of prevalence of non-K1/K2/K5 infection between Chinese and non-Chinese (3.8% vs 29.4%; *P* = 0.008 by Fisher’s exact test). (**c**) Comparison of prevalence of type 2 diabetes between Chinese and non-Chinese (34% vs 88.2%; *P* = 0.0001 by Fisher’s exact test).

The Chinese (*n* = 53) in our patient cohort had a significantly higher probability of infection with K1 compared to the non-Chinese (*n* = 17) consisting of Malays, Indian and Caucasian (71.7% vs 41.2%; *P* = 0.040; Fig. 3a). Chinese were rarely infected with the uncommon KLA capsule types (i.e. non-K1/K2/K5), unlike non-Chinese who were infected with numerous non-K1/K2/K5 isolates including K16, K28, K57, K63 and novel *cps* isolates (3.8% vs 29.4%; *P* = 0.008; Fig. 3b). While only 34% of the Chinese had underlying type 2 diabetes, 88.2% of the non-Chinese were diabetic (*P* = 0.0001; Fig. 3c). K1 strains are known to be capable of causing KLA in healthy individuals due to its increased virulence potential (see results above), whereas non-K1 strains tend to be restricted to KLA patients with predisposing conditions such as diabetes mellitus^3^. Therefore, the overrepresentation of non-K1/K2/K5 *K. pneumoniae* KLA among non-Chinese patients can in part be explained by the higher rate of diabetes within this group.

### Virulence potential of K1 versus non-K1 isolates upon intraperitoneal injection into C57BL/6J mice

**Figure 4:**
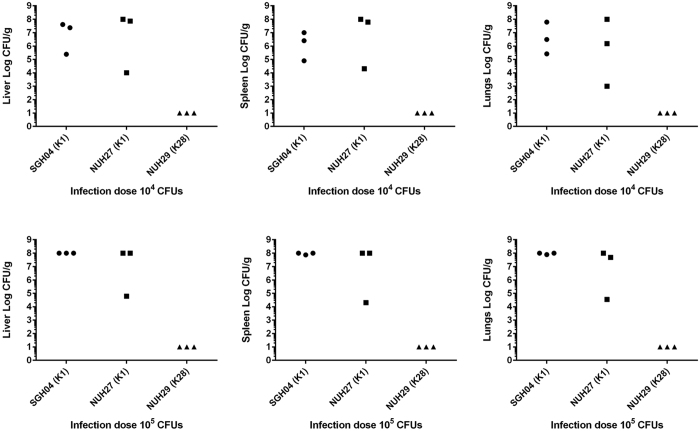
The K1 strains SGH04 and NUH27 were more virulent than the non-K1 strain NUH29. Bacterial burden in the organs of mice upon intraperitoneal injection with 10^4^ or 10^5^ CFUs of SGH04, NUH27 and NUH29. Each dot represents one infected mouse, whose liver, spleen and lungs were harvested 24 hours post-infection. Tissue homogenates that yielded no colonies were plotted with the value 10 CFU/g, which is the approximate limit of detection. The upper limit of quantification is 10^8^ CFU/g.

To verify that K1 isolates are more virulent than non-K1 isolates, we selected representative isolates for mouse infection, where variation of host susceptibility factors is significantly reduced. Two representative K1 strains of different phenotypic profiles were chosen: the highly serum-resistant strain SGH04 and the highly serum-susceptible strain NUH27. SGH04 and NUH27 are clonally related (share the same ST) and possess similar virulence gene profiles: two copies of *rmpA* as well as the *kfu-iuc-iro-irp* clusters. In contrast, the K28 strain NUH29, which represents a distinct ST, lacked *rmpA* and the *kfu-iuc-iro-irp* clusters, although it was highly serum resistant. We chose this isolate as the representative non-K1 strain. Upon intraperitoneal injection of 10^4^ or 10^5^ colony-forming units (CFUs) of SGH04 and NUH27, the majority of the mice became severely morbid after 24 hours post-infection. Consistent with this clinical observation, there was comparably high bacterial burden in the liver, spleen and lungs of these SGH04- and NUH27-infected mice (Fig. 4). In contrast, all the NUH29-infected mice remained healthy and appeared to have cleared the infection at the doses of 10^4^ and 10^5^ CFUs by 24 hours post-infection, since no bacterial load could be detected in their livers, spleens or lungs (Fig. 4). Thus, the difference in *in vivo* virulence between the K1 and non-K1 strains is striking, and highlights the important roles of *rmpA* and the *kfu-iuc-iro-irp* clusters in the virulence of hvKP.

### Susceptibility to extraintestinal infection upon oral inoculation of K1 into healthy control mice versus obese/type 2 diabetic mice

**Figure 5:**
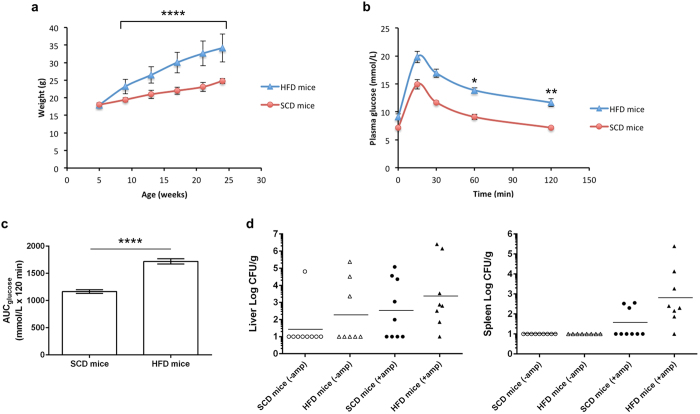
Incidence of extraintestinal infection was not significantly different between obese/glucose intolerant mice and healthy mice upon oral inoculation with hvKP K1. Data in (**a–c**) are means ± SEM. (**a**) Weight of mice fed a SCD or HFD over a course of 19 weeks. Ampicillin treatment between weeks 16 to 19 did not alter body mass. (**b**) Plasma glucose concentrations during the OGTT following 6 hours of fasting in mice fed a SCD or HFD for 16 weeks. Percentage change from basal (fasting glucose level at 0 min) in SCD-fed mice: 15 min = + 107.6% ± 10.0, 30 min = + 64.0% ± 6.3, 60 min = 26.7% ± 5.5, 120 min = + 1.1% ± 5.3. Percentage change from basal in HFD-fed mice: 15 min = + 121.2% ± 15.8, 30 min = + 88.8% ± 12.3, 60 min = 54.9% ± 8.8, 120 min = + 28.5% ± 5.4. Glucose elimination is significantly faster in SCD-fed than HFD-fed mice (*P* = 0.012 and 0.003 by Student’s *t* test, when comparing percentage change at 0–60 minutes and 0–120 minutes, respectively). (**c**) Area under the curve for glucose in (**b**), calculated using the trapezoidal rule. (**d**) Bacterial burden in the extraintestinal organs of SCD-fed (healthy) and HFD-fed (obese/glucose intolerant) mice that were treated with ampicillin or not, upon oral infection with 10^8^ CFUs of the K1 strain SGH04. Each dot represents one infected mouse, whose liver and spleen were harvested 72 hours post-infection. Horizontal bars indicate geometric means. Tissue homogenates that yielded no colonies were plotted with the value 10 CFU/g, which is the approximate limit of detection.

Our patient data had shown that K1 strains caused KLA in predominantly non-diabetic Chinese, suggesting inherent virulence in healthy individuals. We next investigated whether a K1 strain could cause similar disease in healthy mice versus a model of type 2 diabetes in mice^33^,^34^, through oral inoculation which represents a more natural route of infection. Mice on a high-fat diet (HFD) progressively gained more weight than mice on a standard chow diet (SCD) (Fig. 5a). By week 16 of diet feeding, HFD-fed mice were severely obese, with a mean weight 41.1% greater than SCD-fed mice (32.6 ± 3.5 vs 23.1 ± 1.3 g; *P* < 0.0001). HFD-fed mice also progressively developed glucose intolerance, as determined by oral glucose tolerance tests (OGTTs) performed at week 8, 12 and 16 (representative week-16 OGTT shown in Fig. 5b). By week 16, basal blood glucose levels were significantly higher in HFD-fed mice compared to their SCD-fed counterparts following 6 hours of fasting (9.1 ± 0.5 vs 7.2 ± 0.2 mmol/L; *P = *0.002). Upon administration of a bolus of glucose following the 6-hour fast, HFD-fed mice displayed impaired ability to clear glucose in the blood compared to SCD-fed mice. Mean peak blood glucose was reached 15 minutes after glucose challenge in both HFD-fed (19.8 ± 1.0 mmol/L) and SCD-fed mice (14.9 ± 0.8 mmol/L), and was completely cleared over the remaining 105 minutes in SCD-fed mice (7.2 ± 0.2 mmol/L) but only partially eliminated in HFD-fed mice (11.7 ± 0.7 mmol/L) (*P* = 0.003, when comparing the percentage change at 0 and 120 minutes). This marked difference in glucose tolerance between HFD-fed and SCD-fed mice was also apparent with the area under the curve (AUC) measurement (1719.8 ± 48.2 vs 1165.1 ± 31.9 AUC_glucose_ mmol/L × 120 minutes; *P* < 0.0001) (Fig. 5c).

Following the OGTT at week 16, the HFD-fed and SCD-fed mice were further randomized into two groups. One group received sterile drinking water while the other received ampicillin water for three weeks^35^. We chose SGH04 as the prototype K1 strain for the oral infection at week 19 (Fig. 5d). The incidence rate of extraintestinal infection, as determined by bacterial burden in the liver and spleen 72 hours post-infection, is summarized in Table 3. In the no antibiotic treatment group, the incidence of extraintestinal infection was slightly higher in HFD-fed (37.5%, 3/8) compared to SCD-fed mice (11.1%, 1/9), but this difference is not statistically significant (*P* = 0.294). Expectedly, ampicillin treatment significantly increased the incidence of extraintestinal infection as opposed to no antibiotic treatment [12/17 (70.6%) vs 4/17 (23.5%); *P* = 0.015]. Within the ampicillin treatment group, the incidence of extraintestinal infection was also slightly higher in HFD-fed (87.5%, 7/8) compared to SCD-fed mice (55.6%, 5/9). However, this difference is not statistically significant (*P* = 0.294). Moreover, once extraintestinal infection was established, the average bacterial loads in the liver or spleen were comparable between HFD-fed and SCD-fed mice (*P* = 0.755 or *P* > 0.999, respectively).

## Discussion

Our study reports the association between virulence factors of hvKP and demographics and susceptibility factors of KLA patients. We found that the most virulent and widespread capsule type K1 infected the Chinese in our patient cohort at a significantly higher frequency than the non-Chinese (Malays, Indian and Caucasian). One plausible explanation for this finding could be that there is a higher intestinal carriage rate of capsule type K1 in the Chinese compared to the non-Chinese, perhaps occurring from a different environmental source of acquisition. Studies in other extraintestinal pathogenic Enterobacteriaceae such as *E. coli* suggest that the vehicles for strain acquisition are probably through a combination of food, water or person-to-person transmission such as close contact with family members^36^. Indeed, one study in Japan presented evidence of familial spread of hvKP ST23-K1, which caused KLA in two family members at different times whereas the third member was an asymptomatic carrier^37^. This same virulent ST23-K1 clone had been maintained among the Japanese family members for at least two years, supporting the concept that intestinal colonization is requisite for, but does not necessarily lead to clinical disease^37^.

In South Korea, the fecal carriage rate of *K. pneumoniae* in healthy individuals was found to be ~21% (248 positive stool samples out of 1,174), of which 23% were capsule type K1^38^. In contrast, the fecal carriage rate of *K. pneumoniae* in healthy Chinese individuals in Taiwan, Hong Kong, China, Singapore, Malaysia, Japan, Thailand and Vietnam was found to be ~62% (592 out of 954)^39^. Capsule types K1/K2 accounted for ~10% in all the countries surveyed except Thailand and Vietnam, where K1-associated KLA has never been reported^39^. Together, these seroepidemiology studies imply that host genetic factors in combination with environmental factors modulate gut carriage of the various capsule types, to influence KLA development.

Our study also indicates that there are other factors influencing susceptibility to KLA between the various races beyond the dominant effect of K1 infection in Chinese. For example, Indians appear less prone to develop KLA than Malays or Chinese. Although the Indian community has the highest prevalence of diabetes mellitus (an established risk factor for KLA) in Singapore^28^, the Indian race was still under-represented in KLA prevalence based on the population demographics. It will be informative to determine if this under-representation can be validated with a larger cohort, and whether it is due to differences in transmission and carriage rate, or due to host genetics. Therefore, it is crucial to determine the carriage of *K. pneumoniae* and the corresponding capsule types in the intestinal tracts of the various ethnic groups, particularly in Indians, Malays and Caucasians. Data on intestinal carriage of *K. pneumoniae* in non-Chinese is currently very scarce. In addition, unique cultural practices and the food sources regularly consumed by each ethnic group, which is likely to influence intestinal colonization, need to be thoroughly examined to identify the environmental reservoir of the different typeable strains.

The Chinese in our patient cohort were mostly non-diabetic, whereas the vast majority of non-Chinese had underlying type 2 diabetes. Consistent with this, Fang *et al.* had previously reported that K1 strains are capable of causing KLA in healthy individuals with no significant medical histories, whereas non-K1 (particularly, non-K1/K2) strains tend to cause disease in patients with predisposing conditions such as diabetes mellitus^3^. Our mouse infection experiments showed that representative K1 isolates were more virulent than a representative non-K1 isolate, similar to the findings of previous studies^40^. Furthermore, our genotypic data revealed that K1 isolates were more commonly associated with acquired virulence genes than non-K1 isolates, supporting the hypothesis that the former have an increased virulence potential, which presumably facilitates disease development among otherwise healthy individuals.

Lin *et al.* had reported that streptozocin-induced type 1 diabetic mice were more prone to develop extraintestinal infection than naive mice upon oral infection with the K2 strain CG43^41^. Our diet-induced obesity-dependent mouse model mimics type 2 diabetes and is more representative of the clinical profile of most KLA patients. In our model, the incidence rate of extraintestinal infection caused by a representative K1 strain was not significantly higher in obese/glucose intolerant mice than in control mice. This result supports the notion that K1 isolates can cause KLA independent of underlying diseases in the host^3^, and is also consistent with our clinical finding that most of the K1 KLA cases occurred in Chinese patients who did not have type 2 diabetes.

In conclusion, we show that KLA infections in multi-ethnic Singapore fall into two groups: 1) genetically homogenous, hypervirulent K1 capsule type in non-diabetic Chinese, and 2) genetically heterogeneous non-K1 capsule types in diabetic non-Chinese. Our work paves the way for future investigation into the unique transmission patterns and/or genetic predisposition of the various races in driving the epidemiology of KLA.

## Methods

### Recruitment of participants and collection of demographics and clinical information

Study participants were prospectively recruited as part of the A-KLASS clinical trial across three academic medical centers in Singapore^26^. Inclusion criteria include: inpatients, age ≥ 21 years, abdominal imaging suggestive of liver abscess and positive blood or abscess fluid culture for *K. pneumoniae*. Exclusion criteria include: polymicrobial liver abscess, endophthalmitis, central nervous system abscess or severe sepsis at the screening. Information collected at screening include: age, sex, ethnicity, presence of comorbidities, and results of abdominal imaging.

### PCR analysis

*K. pneumoniae* capsule type was determined by allele-specific PCR amplification of the *cps* gene cluster at the *wzy* and *wzx* loci^3^; for non-discriminatory strains of *wzy/wzx-*typing, *wzi* sequencing was performed^42^. Additionally, genotyping for the presence of key virulence-associated genes including *kfu, iuc, iro, irp, rmpA* and *allS* was performed^3^,^10^,^12^. *E. coli* DH5α was selected as a negative control. Specific primers used to detect the alleles of the target gene sequences are tabulated in Supplementary Table S2.

### WGS and analysis

Twenty-seven *K. pneumoniae* isolates were subjected to whole-genome shotgun sequencing on the Illumina MiSeq platform. DNA was extracted using GenElute™ bacterial genomic DNA kit protocol (Sigma-Aldrich). Libraries were constructed using Nextera XT kits and 150-bp sequence reads were generated. Reads were filtered to remove sequences with a mean Phred quality score < 30. Genomes were assembled *de novo* using Spades version 3.6.0^43^. *In silico* MLST, *wzi* allele typing and virulence gene screening was performed using SRST2^44^ and *K. pneumoniae* BIGS database (http://bigsdb.web.pasteur.fr/klebsiella/)^29^. Novel MLST and *wzi* allele sequences were submitted to the *K. pneumoniae* BIGS database for assignment of allele numbers following extraction from *de novo* assembled contigs. The *wzi* allele assignments were checked for associations with known capsule types and compared with PCR typing results. Where neither PCR typing nor *wzi* alleles could be matched to a known capsule type (*n* = 2), BLASTn searches were used to locate the conserved *galF* and *ugd* flanking genes to extract the putative capsule loci (i.e. sequence between the flanking genes) from the assemblies as described previously^45^. BLASTn searches were used to compare the relevant assembled contigs to the 79 known complete Klebsiella capsular locus sequences^9^, NCBI Genbank database and *K. pneumoniae* BIGS database.

### String test for hypermucoviscosity

String test to determine bacterial hypermucoviscosity was performed as described previously^5^. Briefly, a bacteriologic loop was used to stretch a mucoviscous string from a second subculture of *K. pneumoniae* colony on blood agar (Sigma-Aldrich). Hypermucoviscosity is semi-quantitatively defined by the formation of viscous strings > 5 mm in length.

### Serum resistance assays

Bacterial susceptibility to healthy pooled human serum was determined by the method of Podschun *et al.*^46^, with slight modifications. Briefly, bacterial strains were diluted to 1 × 10^6^ CFU/mL in PBS. Next, 25 uL of bacterial suspension was added to 75 uL of pooled healthy human serum (Sigma-Aldrich). Viability was determined immediately and after 3 hours of incubation at 37 °C by plating out serial dilutions on LB agar. Responses were graded as follows: grade 5 (highly serum-resistant), viable CFU after 3 hours of incubation in serum > 100% of the inoculum; grade 4 (serum-resistant), 71–100%; grade 2 (serum-susceptible), 1–30%; grade 1 (highly serum-susceptible), 0%. All assays were repeated at least three times.

### Mouse model for intraperitoneal injection with *K. pneumoniae*

Eight-week-old C57BL/6J mice (*n* = 18) were infected with the K1 strains SGH04 and NUH27 or the K28 strain NUH29 via intraperitoneal injection at doses of 10^4^ and 10^5^ CFUs in 100 μL PBS. Mice were euthanized 24 hours post-infection, and liver, spleen and lungs were harvested to determine bacterial burden by plating serial dilutions on HiCrome™ Klebsiella selective agar (Sigma-Aldrich).

### Type 2 diabetes mouse model for oral infection with *K. pneumoniae*

Five-week-old C57BL/6 J mice (*n* = 34) were fed *ad libitum* with either SCD or HFD containing 35% lard (Harlan Teklad, Cat# TD.03584) under specific pathogen-free conditions. Following 8, 12 and 16 weeks of diet feeding, OGTTs were performed. After the final OGTT at week 16, the SCD-fed and HFD-fed mice were each randomized into two groups: the first group received normal untreated drinking water while the second group were administered with clinical-grade ampicillin (Sandoz, 1 g/L) in the drinking water for three weeks. Upon cessation of antibiotic treatment, mice were allowed to recover for 24 hours (i.e. ampicillin water replaced with normal drinking water). All 24-week-old mice were then orally infected with 10^8^ CFUs of the K1 strain SGH04 in 100 μL PBS using a 20-gauge, 38 mm length, flexible plastic feeding tube with soft elastomer tip (Prime Bioscience). Mice were euthanized 72 hours post-infection, and liver and spleen were harvested to determine bacterial burden by plating serial dilutions on HiCrome™ Klebsiella selective agar (Sigma-Aldrich).

### OGTTs

OGTTs were performed according to the recommendations by Andrikopoulos *et al.*^47^. Briefly, mice were fasted for six hours prior to administration with 2 g/kg glucose into the stomach via oral gavage using a 20-gauge, 38 mm length, flexible plastic feeding tube with soft elastomer tip (Prime Bioscience). Blood was drawn at 0, 15, 30, 60 and 120 minutes post glucose challenge by puncturing the lateral tail vein, and blood glucose was measured using Accu-Chek^®^ Performa glucose meter and test strips (Roche).

### Ethics statement

The A-KLASS protocol and the associated informed consent documents were reviewed and approved by National Healthcare Group Domain Specific Review Board, SingHealth Centralized Institutional Review Board and Health Science Authority. Study procedures were conducted in accordance with the approved guidelines and regulations, and written informed consent was obtained from all human participants. The animal protocols were reviewed, approved and carried out in strict accordance to the recommendations by Institutional Animal Care and Use Committee from the National University of Singapore.

### Statistical analysis

Statistical tests were performed using GraphPad Prism 6, with the use of Fisher’s exact test for dichotomous data, Student’s *t* test for normally distributed continuous data and Mann-Whitney U test for non-normally distributed continuous data. *P* value of <0.05 was considered statistically significant.

## Additional Information

**How to cite this article**: Lee, I. R. *et al.* Differential host susceptibility and bacterial virulence factors driving Klebsiella liver abscess in an ethnically diverse population. *Sci. Rep.* **6**, 29316; doi: 10.1038/srep29316 (2016).

## Supplementary Material

Supplementary Information

## Figures and Tables

**Table 1:** KLA patient characteristics (*n* = 70).

Variable	Mean / Frequency
Demographics	
Age, mean years ± SD (range)	59.8 ± 11.9 (30–86)
Male gender	56/70 (80.0%)
Ethnicity	
Chinese	53/70 (75.7%)
Malay	15/70 (21.4%)
Indian	1/70 (1.4%)
Others	1/70 (1.4%)
Underlying disease	
Type 2 diabetes mellitus	33/70 (47.1%)
Cardiovascular disease	29/70 (41.4%)
Hypertension	24/70 (34.3%)
Hyperlipidemia	22/70 (31.4%)
Gastrointestinal tract disease	11/70 (15.7%)
Anaemia	10/70 (14.3%)
Biliary tract disease	9/70 (12.9%)
Neurologic disease	7/70 (10%)
Pulmonary disease	5/70 (7.1%)
Chronic kidney disease	5/70 (7.1%)
Hepatic disease	5/70 (7.1%)
Malignancy	4/70 (5.7%)
Hepatitis B	4/70 (5.7%)
Gallstones	2/70 (2.9%)
Radiological imaging	
Size of liver abscess, mean mm ± SD (range)	57.6 ± 30.6 (10–140)
Multiple liver abscesses	28/70 (40.0%)

**Table 2:** Genotypic and phenotypic virulence profiles of the KLA clinical isolates (*n* = 70).

Strain name	Capsule type	ST	HVP	*rmpA*copy number	*allS*	*kfu*	*iuc*	*irp*	*iro*	Serum resistance grade
TTSH01	K1	NT	+	2	+	+	+	+	+	5
TTSH02	K1	NT	+	2	+	+	+	+	+	5
TTSH06	K1	NT	+	2	+	+	+	+	+	5
TTSH07	K1	NT	+	2	+	+	+	+	+	5
TTSH08	K1	NT	+	2	+	+	+	−	+	5
TTSH10	K1	NT	+	2	+	+	+	+	+	5
TTSH11	K1	NT	+	2	+	+	+	+	+	5
TTSH12	K1	NT	+	2	+	+	+	+	+	2
TTSH16	K1	NT	+	2	+	+	+	+	+	5
TTSH17	K1	NT	+	2	+	+	+	+	+	5
TTSH20	K1	NT	+	2	+	+	+	+	+	5
TTSH23	K1	NT	+	2	+	+	+	+	+	4
TTSH24	K1	NT	+	2	+	+	+	+	+	5
TTSH26	K1	NT	+	2	+	+	+	+	+	5
TTSH27	K1	NT	+	2	+	+	+	+	+	2
NUH01	K1	23	+	2	+	+	+	+	+	4
NUH02	K1	23	+	2	+	+	+	+	+	5
NUH05	K1	NT	+	2	+	+	+	+	+	5
NUH06	K1	NT	+	2	+	+	+	+	+	5
NUH07	K1	NT	+	2	+	+	+	−	+	5
NUH08	K1	23	+	2	+	+	+	+	+	5
NUH12	K1	NT	+	2	+	+	+	+	+	5
NUH13	K1	NT	+	2	+	+	+	+	+	4
NUH15	K1	23	+	2	+	+	+	+	+	5
NUH16	K1	23	+	2	+	+	+	+	+	5
NUH17	K1	23	+	2	+	+	+	+	+	5
NUH18	K1	NT	+	2	+	+	+	+	+	5
NUH19	K1	23	+	2	+	+	+	+	+	5
NUH20	K1	NT	+	2	+	+	+	+	+	5
NUH21	K1	NT	+	2	+	+	+	+	+	5
NUH22	K1	NT	+	2	+	+	+	+	+	5
NUH23	K1	23	+	2	+	+	+	+	+	5
NUH24	K1	23	+	2	+	+	+	−	+	5
NUH27	K1	23	+	2	+	+	+	+	+	2
NUH31	K1	NT	+	2	+	+	+	+	+	5
NUH32	K1	NT	+	2	+	+	+	+	+	5
NUH33	K1	NT	+	2	+	+	+	+	+	5
NUH35	K1	NT	+	1	+	+	+	+	+	5
SGH01	K1	NT	+	2	+	+	+	+	+	5
SGH03	K1	NT	+	2	+	+	+	+	+	5
SGH04	K1	23	+	2	+	+	+	+	+	5
SGH05	K1	23	+	2	+	+	+	+	+	5
SGH06	K1	NT	+	2	+	+	+	+	+	5
SGH09	K1	NT	+	2	+	+	+	+	+	5
SGH10	K1	23	+	2	+	+	+	+	+	5
TTSH03	K2	NT	+	2	−	−	+	−	+	5
TTSH05	K2	373	+	1	−	−	−	+	+	5
TTSH09	K2	NT	+	2	−	+	+	+	+	5
TTSH13	K2	380	+	1	−	+	+	−	+	5
TTSH14	K2	NT	+	1	−	+	+	−	+	5
TTSH25	K2	NT	+	1	−	−	+	+	+	5
NUH03	K2	65	+	2	−	−	+	+	+	5
NUH04	K2	2039^a^	−	0	−	−	+	+	+	1
NUH10	K2	NT	+	1	−	−	+	+	+	5
NUH14	K2	2038^a^	+	1	+	+	+	+	+	5
NUH25	K2	NT	+	2	−	−	+	+	+	5
SGH02	K2	NT	+	1	−	−	+	−	+	5
SGH08	K2	NT	+	1	−	−	+	−	+	5
NUH34	K2	NT	+	2	−	+	+	+	+	4
TTSH15	K5	NT	+	1	−	+	+	+	−	5
TTSH18	K5	828	+	2	−	+	+	+	+	5
NUH26	K5	1049	+	2	−	+	+	+	+	5
SGH07	K5	60	+	1	−	+	−	+	+	5
NUH09	K16	660	+	2	−	+	+	+	+	5
NUH29	K28	20	−	0	−	−	−	−	−	5
TTSH19	K57	NT	+	2	−	−	+	−	+	5
NUH28	K57	592	+	1	−	+	+	−	+	2
NUH36	K63	111	+	1	−	−	−	+	+	5
TTSH04	Novel	2037^a^	−	0	−	+	−	−	−	1
NUH11	Novel	399	−	0	−	−	−	−	−	1

Abbreviations: ST, sequence-type; NT, not tested; HVP, hypermucoviscous phenotype

^a^Newly identified in this study

**Table 3:** Incidence of developing extraintestinal hvKP K1 infection in healthy control mice and obese/glucose intolerant mice.

	No antibiotic treatment			Ampicillin treatment		
	SCD-fed mice(*n* = 9)	HFD-fed mice(*n* = 8)	*P*value^b^	SCD-fed mice(*n* = 9)	HFD-fed mice(*n* = 8)	*P*value^b^
Liver	1 (11.1%)	3 (37.5%)	0.294	5 (55.6%)	7 (87.5%)	0.294
Spleen	0 (0%)	0 (0%)	–	3 (33.3%)	7 (87.5%)	0.050
Incidence^a^	1 (11.1%)	3 (37.5%)	0.294	5 (55.6%)	7 (87.5%)	0.294

^a^Incidence of extraintestinal infection was calculated as the percentage of mice that had positive hvKP K1 culture yielded in any of the extraintestinal organs tested.

^b^*P* value was determined by Fisher’s exact test.
