# Supplementary material for: Differential host susceptibility and bacterial virulence factors driving Klebsiella liver abscess in an ethnically diverse population
Source: Sci Rep. 2016 Jul 13;6:29316. doi: 10.1038/srep29316 (PMC4942785; doi:10.1038/srep29316)
Supplement: Supplementary Information [file srep29316-s1.doc]

**Differential host susceptibility and bacterial virulence factors driving Klebsiella liver abscess in an ethnically diverse population**

I. Russel Lee^1^, James S. Molton^2,3^, Kelly L. Wyres^4,5^, Claire Gorrie^4,5^, Jocelyn Wong^1^, Chu Han Hoh^1^, Jeanette Teo^6^, Shirin Kalimuddin^7^, David C. Lye^3,8^, Sophia Archuleta^2,3^, Kathryn E. Holt^4,5^ & Yunn-Hwen Gan^1^

^1^ Department of Biochemistry, Yong Loo Lin School of Medicine, National University of Singapore

^2^ Division of Infectious Diseases, University Medicine Cluster, National University Health System, Singapore

^3^ Department of Medicine, Yong Loo Lin School of Medicine, National University of Singapore

^4^ Centre for Systems Genomics, University of Melbourne, Parkville, Victoria 3010, Australia

^5^ Department of Biochemistry and Molecular Biology, Bio21 Molecular Science and Biotechnology Institute, University of Melbourne, Parkville, Victoria 3010, Australia

^6^ Department of Laboratory Medicine, Microbiology Unit, National University Hospital, Singapore

^7^ Department of Infectious Diseases, Singapore General Hospital, Singapore

^8^ Communicable Disease Center, Institute of Infectious Diseases and Epidemiology, Tan Tock Seng Hospital, Singapore

**Supplementary Table S1.** *In silico* MLST of seven housekeeping genes and *wzi* allele typing derived from WGS of the KLA isolates (*n* = 27).

| **Strain name** | **Capsule type** | **ST** | ***gapA*** | ***infB*** | ***mdh*** | ***pgi*** | ***phoE*** | ***rpoB*** | ***tonB*** | ***wzi* allele** |
| --- | --- | --- | --- | --- | --- | --- | --- | --- | --- | --- |
| NUH01 | K1 | 23 | 2 | 1 | 1 | 1 | 9 | 4 | 12 | 1 |
| NUH02 | K1 | 23 | 2 | 1 | 1 | 1 | 9 | 4 | 12 | 1 |
| NUH08 | K1 | 23 | 2 | 1 | 1 | 1 | 9 | 4 | 12 | 1 |
| NUH15 | K1 | 23 | 2 | 1 | 1 | 1 | 9 | 4 | 12 | 1 |
| NUH16 | K1 | 23 | 2 | 1 | 1 | 1 | 9 | 4 | 12 | 1 |
| NUH17 | K1 | 23 | 2 | 1 | 1 | 1 | 9 | 4 | 12 | 1 |
| NUH19 | K1 | 23 | 2 | 1 | 1 | 1 | 9 | 4 | 12 | 1 |
| NUH23 | K1 | 23 | 2 | 1 | 1 | 1 | 9 | 4 | 12 | 1 |
| NUH24 | K1 | 23 | 2 | 1 | 1 | 1 | 9 | 4 | 12 | 1 |
| NUH27 | K1 | 23 | 2 | 1 | 1 | 1 | 9 | 4 | 12 | 1 |
| SGH04 | K1 | 23 | 2 | 1 | 1 | 1 | 9 | 4 | 12 | 1 |
| SGH05 | K1 | 23 | 2 | 1 | 1 | 1 | 9 | 4 | 12 | 1 |
| SGH10 | K1 | 23 | 2 | 1 | 1 | 1 | 9 | 4 | 12 | 1 |
| TTSH05 | K2 | 373 | 9 | 4 | 2 | 26 | 1 | 1 | 27 | 2 |
| TTSH13 | K2 | 380 | 2 | 1 | 1 | 1 | 1 | 4 | 19 | 203 |
| NUH03 | K2 | 65 | 2 | 1 | 2 | 1 | 10 | 4 | 13 | 72 |
| NUH04 | K2 | 2039^a^ | 2 | 3 | 2 | 4 | 9 | 4 | 13 | 205 |
| NUH14 | K2 | 2038^a^ | 2 | 1 | 1 | 1 | 1 | 4 | 36 | 203 |
| TTSH18 | K5 | 828 | 2 | 1 | 1 | 26 | 1 | 1 | 54 | 5 |
| NUH26 | K5 | 1049 | 2 | 3 | 4 | 97 | 12 | 1 | 39 | 208 |
| SGH07 | K5 | 60 | 2 | 1 | 2 | 1 | 4 | 4 | 8 | 5 |
| NUH09 | K16 | 660 | 2 | 1 | 2 | 1 | 4 | 1 | 25 | 16 |
| NUH29 | K28 | 20 | 2 | 3 | 1 | 1 | 4 | 4 | 4 | 84 |
| NUH28 | K57 | 592 | 2 | 3 | 6 | 1 | 9 | 4 | 13 | 206 |
| NUH36 | K63 | 111 | 2 | 1 | 5 | 1 | 17 | 4 | 42 | 63 |
| TTSH04 | Novel | 2037^a^ | 18 | 22 | 26 | 61 | 31 | 119 | 51 | 207 |
| NUH11 | Novel | 399 | 2 | 1 | 11 | 1 | 10 | 10 | 12 | 123 |

^a^ Newly identified sequence-type

**Supplementary Table S2.** Diagnostic primers used in this study.

| **Primer name** | **Target gene in *K. pneumoniae*** | **Sequence (5’- 3’)** |
| --- | --- | --- |
| RL primer 1 | K1 *magA* | GGTGCTCTTTACATCATTGC |
| RL primer 2 | K1 *magA* | GCAATGGCCATTTGCGTTAG |
| RL primer 3 | K1 *wzx* | GTAGGTATTGCAAGCCATGC |
| RL primer 4 | K1 *wzx* | GCCCAGGTTAATGAATCCGT |
| RL primer 5 | K2 *wzy* | GGATTATGACAGCCTCTCCT |
| RL primer 6 | K2 *wzy* | CGACTTGGTCCCAACAGTTT |
| RL primer 7 | K2 *wzx* | GGAGCCATTTGAATTCGGTG |
| RL primer 8 | K2 *wzx* | TCCCTAGCACTGGCTTAAGT |
| RL primer 9 | K5 *wzy* | CAGGGAACTCCTACGCAGATTT |
| RL primer 10 | K5 *wzy* | GGGTGATAAGGTATAGCTGACAC |
| RL primer 11 | K5 *wzx* | GCCACCTCTAAGCATATAGC |
| RL primer 12 | K5 *wzx* | CGCACCAGTAATTCCAACAG |
| RL primer 25 | K20 *wzy* | CGGTGCTACAGTGCATCATT |
| RL primer 26 | K20 *wzy* | GTTATACGATGCTCAGTCGC |
| RL primer 27 | K20 *wzx* | CCGATTCGGTCAACTAGCTT |
| RL primer 28 | K20 *wzx* | GCACCTCTATGAACTTTCAG |
| RL primer 29 | K54 *wzy* | GTTACCTCAGAGCGTTGCAT |
| RL primer 30 | K54 *wzy* | CGGACTTAATAGCGAGCAAAG |
| RL primer 31 | K54 *wzx* | CATTAGCTCAGTGGTTGGCT |
| RL primer 32 | K54 *wzx* | GCTTGACAAACACCATAGCAG |
| RL primer 33 | K57 *wzy* | CTCAGGGCTAGAAGTGTCAT |
| RL primer 34 | K57 *wzy* | CACTAACCCAGAAAGTCGAG |
| RL primer 35 | K57 *wzx* | CGACAAATCTCTCCTGACGA |
| RL primer 36 | K57 *wzx* | CGCGACAAACATAACACTCG |
| RL primer 37 | Plasmid *rmpA* | TACTTTATATGTAACAAGGATGTAAACATAG |
| RL primer 38 | Plasmid *rmpA* | CAGTAGGCATTGCAGCACTGC |
| RL primer 39 | Plasmid *rmpA2* | CTGTGTCCACTATTGGTGGG |
| RL primer 40 | Plasmid *rmpA2* | GATAGTTCACCTCCTCCTCC |
| RL primer 41 | Chromosomal *rmpA* | TGGCAGCAGGCAATATTGTC |
| RL primer 42 | Chromosomal *rmpA* | GAAAGAGTGCTTTCACCCCCT |
| RL primer 19 | *allS* | CCGAAACATTACGCACCTTT |
| RL primer 20 | *allS* | ATCACGAAGAGCCAGGTCAC |
| RL primer 21 | *kfu* | ATAGTAGGCGAGCACCGAGA |
| RL primer 22 | *kfu* | AGAACCTTCCTCGCTGAACA |
| RL primer 15 | *iuc* | GCATAGGCGGATACGAACAT |
| RL primer 16 | *iuc* | CACAGGGCAATTGCTTACCT |
| RL primer 43 | *irp* | GATGGCACAATCGAGTTCCT |
| RL primer 44 | *irp* | GCCCGACATACTCAATCTGT |
| RL primer 45 | *iro* | GTCCGGCGGTAACTTCAGCC |
| RL primer 46 | *iro* | TCAGAATGAAACTACCGCCC |
